# Effect of Novel Bacteriocinogenic *Lactobacillus fermentum* BZ532 on Microbiological Shelf-Life and Physicochemical and Organoleptic Properties of Fresh Home-Made Bozai

**DOI:** 10.3390/foods10092120

**Published:** 2021-09-08

**Authors:** Hafiz Abdul Rasheed, Tuhanguli Tuoheti, Zhiyu Li, Mekonen Tekliye, Yongzhu Zhang, Mingsheng Dong

**Affiliations:** College of Food Science and Technology, Nanjing Agricultural University, 1 Weigang Road, Nanjing 210095, China; harasheed39@gmail.com (H.A.R.); tuhangul622@126.com (T.T.); lizhiyu1008@163.com (Z.L.); cherinet2018@gmail.com (M.T.); 2016208018@njau.edu.cn (Y.Z.)

**Keywords:** bozai (Boza), *Lactobacillus fermentum* BZ532, co-culture, bacteriocin LF-BZ532, *Staphylococcus aureus*, *Escherichia coli* k-12

## Abstract

Bacteriocinogenic *Lactobacillus fermentum* BZ532 with novel bacteriocin LF-BZ532 was originally isolated from Chinese cereal fermented drink, showing an antimicrobial characteristic during fermentation. This study aimed to explore the in situ antimicrobial activities of *L. fermentum* BZ532 and co-culturing investigation against key food pathogens, i.e., *Staphylococcus aureus* and *Escherichia coli* K-12, was conducted during fresh bozai production. The growth of spoilage bacteria was suppressed and bacterial count was reduced to a significantly low level during 48 h of co-cultures. In situ production of antimicrobial compounds expressed positive activity against *S. aureus* and *E. coli* K-12, but negative acitivity against *Salmonella* sp. D104. The total viable count of bozai BZ-*Lf* (bozai fermented with BZ532 strain) had a comparatively lower viable count than bozai BZ-*C* (bozai as an experimental control without BZ532) during storage of 7 days. Titratable acidity of bozai treatments (BZ-*C*, BZ-*Lf*) was increased, while pH declined accordingly during storage of 7 days. The organoleptic quality of bozai BZ-*C* had low sensorial scores as compared with BZ-*Lf* during storage. In comparison with naturally fermented bozai (BZ-*C*), *L. fermentum* BZ532 (BZ-*Lf*) could significantly reduce the microbial spoilage and extend the shelf-life based on microbiological examination. Conclusively, *L. fermentum* BZ532 can be used as a bio-protective culture for improving the safety of bozai.

## 1. Introduction

Microbes compete for the nutrients and limited space existing in natural ecological niches. Consequently, they have developed numerous approaches to survive; production of antimicrobial compounds like bacteriocins is one of them. Bacteriocins are antimicrobial peptides/proteins synthesized ribosomally broadly dispersed in nature [1]. This peptide biodiversity is supported by numerous differences in its characterization and structures. The application of purified or semi-purified bacteriocins or bacteriocinogenic lactic acid bacteria (LAB) as protective cultures is one technological alternative to traditional preservation strategies (i.e., chemical additives). Bacteriocins are considered the most appropriate substitutes to synthetic preservatives due to their non-toxic nature to eukaryotic cells [1].

Among bacteriocinogenic lactic acid bacteria, most of the produced bacteriocins have been isolated mainly from the genus Lactobacillus, because of the diversity of its species and habitats [2]. Some findings have focused on bacteriocin production by strains of *Lactobacillus fermentum* (*L. fermentum*) and *Lactobacillus salivarius* (*L. salivarius*), which are the predominant lactobacilli in human milk and display interesting probiotic and inhibitory properties [3,4].

Several strains of *L. fermentum* were isolated from several sources, such as conventionally fermented milk [5]. It has been one of the specific predominant lactobacilli in the human intestine tract [6,7]. A former clinical study exhibited that *L. fermentum* RC-14 played a significant role in controlling the stability of microflora [8]. Screening for potent probiotic characteristics of *L. fermentum* isolated from different traditional milk products. Prior studies supported that some *L. fermentum* shows probiotic characteristics and is a possible probiotic candidate for gastrointestinal tract-related problems [9,10]. *L. fermentum* secreted inhibitory substances, including bacteriocins, bio-surfactants, and H_2_O_2_, to inhibit the growth of urogenital and intestinal pathogens [10,11]. The clinical findings revealed that *L. fermentum* was effective in reducing intestinal pathogenic microbes and increasing the ratio of probiotic bacteria in healthy individuals [11]. *L. fermentum* antimicrobial attributes and specific production of antimicrobial compounds (called fermenticins) are described as potential possible means for food preservation and medical application [12]. The genus *Lactobacillus* and species *L. fermentum* have been subjected to a safety risk assessment under the scientific committee of the European Food Safety Authority (EFSA) and approved on the QPS-recommended list (qualified presumption of safety) and certified to be applied to different food systems [13].

The World Health Organization (WHO) designates that *Campylobacter,* enterohemorrhagic *Escherichia coli (E. coli), Salmonella, Listeria monocytogenes (L. monocytogenes*), and *Vibrio cholerae* are among the most common foodborne bacteria that severely affect millions of people around the globe [14]. Milk and cereal products that are not produced with appropriate hygienic conditions can be contaminated with food pathogens like *L. monocytogenes, Campylobacter jejuni*, *Salmonella* spp., and *E. coli* O157:H7, causing foodborne outbreaks. Among others, *E. coli* is a foodborne pathogen of major concern for the cereal and dairy industry. Most strains of *E. coli* remain non-pathogenic in the intestinal tract. However, some strains may cause infections in the urinary tract, gastroenteritis, meningitis, and septicemia. *E. coli* O157:H7, which belongs to the enterohemorrhagic *E. coli* (EHEC) class, is the most pathogenic serotype of *E. coli* [14].

The appropriate hygiene applications are compulsory to reduce potential contamination during food production. However, good manufacturing practices may not be sufficient to ensure the safety of food products. In this approach, the food industry has been adopting alternatives to decrease contamination and regulate the growth of microbes that may reach food during the production chain, such as bacteriocins secreted by lactic acid bacteria [15]. In previous and current decades, studies on the usage of bacteriocins indicate that they can provide several benefits in food products, such as decreasing the risk of proliferation of food spoilage pathogens, extending the shelf-life and safety of the end products, and limiting the use of synthetic chemical preservatives [15,16,17]. Currently, there is some bacteriocin that can be used commercially as a food preservative for the control of foodborne spoilage microorganisms in various food systems, thanks to its GRAS (generally recognized as safe) status; the use of nisin in food production is approved in approximately fifty countries [18,19,20]. These restrictions of alternatives have stimulated numerous researchers to characterize novel bacteriocinogenic strains and their bacteriocins focused on a potential application as food bio-preservatives in different food systems.

The most extensively grown millet species around the globe is pearl millet (*Pennisetum glaucum* L. R. Br.), followed by foxtail (*Setaria italica* L.) [21,22]. In comparison with some other cereals, millet is comprised of a high fiber percentage, mineral composition, and protein quality, which considerably contribute to the nutritional security of the population existing in the millet cultivating regions [23]. Moreover, millet is not only higher in nutritional value, but also superior to the key cereals in terms of protein, vitamins, minerals, and energy [24]. Additionally, millet is recommended as a good source of nutraceuticals, phytochemicals dietary fiber, and micronutrients.

Bozai/Boza is a drink that has been produced from the fermentation of millet, maize, and barley, among others. Cooked cereal is inoculated either with specific microbial culture or previously prepared bozai or sourdough as a starter. The sludge is fermented at 30–37 °C for 24 h and kept at 4 °C for 2–3 days, as reported previously [25,26]. In our previous study, bozai has been explored in terms of bacteriocinogenic nature and the same fermentation method as “Boza” was reported, which is widely reported in different countries [27]. It produces the bacteriocin LF-BZ532, which has been previously characterized [27]. This bacteriocin shows the inhibitory activity against several food pathogens such as *Escherichia coli* K-12 (*E. coli* K-12), *Staphylococcus aureus* ATCC6538 (*S. aureus* ATCC6538), and *Listeria monocytogenes*.

This study aimed to investigate the effect of bacteriocinogenic *Lactobacillus fermentum* BZ532 strain (*L. fermentum* BZ532) on freshly prepared bozai, specifically with regards to co-culturing of BZ532 strain against food spoilage microorganisms, i.e., *E. coli*, *S. aureus*, in situ bacteriocin production evaluation by agar well diffusion assay against key food pathogens, and physicochemical and sensory evaluation with and without bacteriocinogenic *L. fermentum* BZ532.

## 2. Materials and Methods

Millet seeds (*Pennisetum glaucum* L. R. Br.) as a raw material to produce fresh bozai were purchased from a local market located in Nanjing, China.

### 2.1. Bacterial Strains and Culture Conditions

*L. fermentum* BZ532 is a bacteriocinogenic strain isolated from the fermented beverage named bozai. *L. fermentum* BZ532 was grown in De Man, Rogosa, and Sharpe (MRS) broth Merk, Darmstadt, Germany) at 37 °C for 24 h, as previously described [27]. *E. coli* K-12, *S. aureus* ATCC6538, and *Salmonella* sp. D104 were used as indicator strains in the present study and activated as reported previously [27].

### 2.2. Fresh Bozai Production

**Figure 1:**
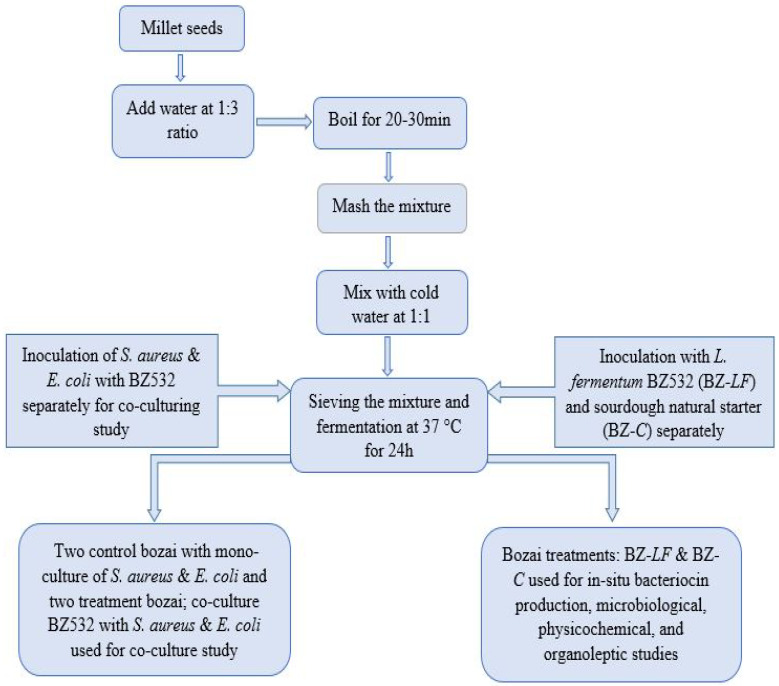
Flow diagram of bozai production.

Millet seeds were purchased from a local market located in Nanjing, China, and immediately delivered to our lab under aseptic conditions. Millet seeds were washed with sterile water and soaked overnight in sterile water. Afterward, they were cooked in boiling water with continuous stirring for 20–30 min. Then, the mixture was homogenized and water was added to reach the required consistency. The mixture was further strained for removing the solid phase and sugar (5%) was added to the liquid phase. Finally, the sweet liquid was inoculated with an overnight culture of *L. fermentum* BZ532 in MRS (10^5^ CFU/mL, 1% *v*/*v*) and incubated for 24 h at 37 °C (Figure 1). Moreover, four bozai treatments, co-culturing the BZ532 strain with two food pathogenic strains (*E. coli* K-12 and *S. aureus* ATCC6538), were prepared separately for co-culturing study; i.e., first, control bozai was inoculated with mono-culture of *S. aureus* and its treatment co-culture the BZ532 strain with *S. aureus* in bozai; Second, control bozai was inoculated with mono-culture of *E. coli* and its treatment co-culture the BZ532 strain with *E. coli*, in order to study their antimicrobial effect. An additional bozai treatment was also produced as an experimental control by natural fermentation (sourdough starter), without the addition of *L. fermentum* BZ532, for physicochemical and organoleptic evaluation. Microbiological (TVC and LAB count), physicochemical, and organoleptic evaluations were performed at 1, 3, 5, and 7 days of storage at 4 °C.

### 2.3. Microbiological Analysis

#### 2.3.1. Total Viable Count (TVC) and LAB Enumeration

The bozai sample of different treatments was procured on storage days 1, 3, 5, and 7 to determine the TVC. In brief, 10 g of bozai sample was homogenized in 90 mL of 0.85% NaCl (*w*/*v*) sterilized saline and ten-fold dilutions were performed with the same saline. Different dilutions were pour plated on plate count agar (PCA; Oxoid), and then incubated at 37 °C for 24 h. The results were expressed as log CFU/g. LAB populations were counted by pour plate of particular dilutions on MRS agar and incubated at 37 °C for 36 h; the colonies were enumerated after a specific incubation time and the results were expressed in CFU/g according to previously reports [28,29].

#### 2.3.2. Co-Culturing of *L. fermentum* BZ532 with Pathogenic Bacteria during Bozai Production

Two food pathogen indicator strains (*S. aureus* and *E. coli* K-12) and *L. fermentum* BZ532 were inoculated simultaneously to produce fresh bozai at a level of 10^4^ CFU/mL of indicator strains and 10^5^ CFU/mL for bacteriocinogenic BZ532, respectively, and then incubated (48 h) at 37 °C. Enumeration of various strains was conducted at specific incubation intervals of 0, 4, 8, 12, 24, 36, and 48 h by pouring specific ten-fold dilution on Baird–Parker agar (BP; Oxoid) for *S. aureus*, violet red bile glucose agar (VRBG; Oxoid, Basingstoke, UK) for *E. coli* K-12, and MRS agar for *L. fermentum* BZ532, and then incubated for 48 h at 37 °C.

#### 2.3.3. In Situ Production of Bacteriocin Antimicrobial Compounds

The bacteriocin LF-BZ532 produced in bozai fermented by the strain *L. fermentum* BZ532 was evaluated according to the method of Castilho et al. [30], with some modifications. Bozai sample (10 g) was homogenized with 10 mL of 0.02 N HCl and centrifuged (12,000× *g*, 15 min, 4 °C). The pH of attained cell-free supernatant (CFS) was set to 6.0 with 4N NaOH and lyophilized. Afterward, 5 µg lyophilized CFS was diluted with 150 μL of Ringer × 1/4 and 40 μL of this CFS was transferred to wells of different test plates inoculated with three indicator strains of *S. aureus* (10^6^ CFU/mL), *E. coli.* K-12 (10^6^ CFU/mL), and *Salmonella* sp. D104 (10^6^ CFU/mL) on BP (0.7% agar), VRBG (0.7% agar), and LB (0.7% agar) medium, respectively. Then, they were incubated at 37 °C for 24 h and inhibition zones were observed to detect the production of bacteriocin in the tested bozai samples.

### 2.4. Physicochemical Evaluation of Bozai

All the treatments of bozai from 1 to 7 days of storage were subjected to physicochemical analysis, i.e., titratable acidity, pH, and protein content according to AOAC methods [31].

#### 2.4.1. Titratable Acidity

The obtained samples of bozai were subjected to potentiometric titration with 0.1 N NaOH up to pH 8 to analyze the total titratable acidity according to AOAC methods with some modifications [31]. All samples including control and treatment groups were evaluated in duplicate and the results were calculated according to the following formula and expressed as % lactic acid.

% acid = [(ml of NaOH used] × [0.1 N NaOH] × milliequivalent factor × 100)]/grams of sample.

#### 2.4.2. pH

The pH was determined using a digital pH meter (PHS-3C, Shanghai, China) according to the method described by [32,33]. In brief, 5 g of bozai sample was diluted in 20 mL of neutral distilled water and homogenized. The pH meter was calibrated against buffer solution (pH 4 and 6.86).

#### 2.4.3. Total Protein Content

The total protein in terms of the crude protein content of bozai samples was determined according to the Kjeldahl method with some modifications and multiplied by a constant factor of 6.25 to quantify the crude protein proportion [32,33].

### 2.5. Organoleptic Evaluation

The organoleptic properties of bozai treatments (BZ-*C*, bozai without *L. fermentum* BZ532 and BZ-*Lf*, bozai fermented with *L. fermentum* BZ532) were evaluated by ten panelists according to the process described by [34] with slight modification. Color, odor, mouthfeel, and overall acceptability of bozai samples were evaluated and scores were assigned on a hedonic scale from 1 (extremely dislike) to 9 (extremely like), as previously reported [34].

### 2.6. Statistical Analysis

All experiments were carried out in triplicate and the result was expressed as a mean ± standard deviations (SD), and all data were compared to check the significance by ANOVA (*p* < 0.05) and Tukey’s *t*-test by software STATISTIX 8.1.

## 3. Results and Discussion

### 3.1. Changes in TVC and L. fermentum BZ532 Enumeration during Bozai Storage

**Figure 2:**
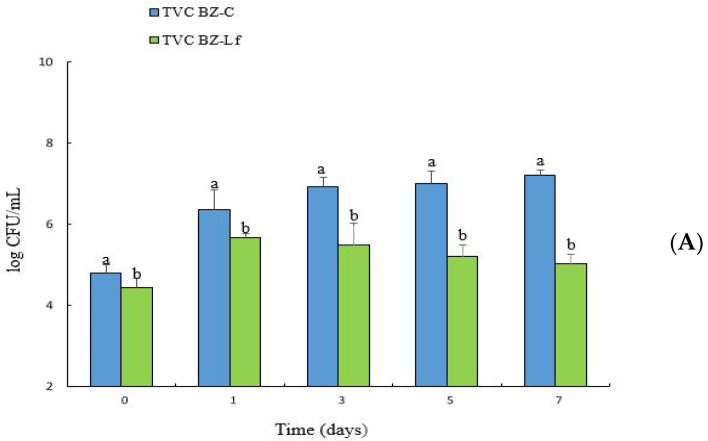

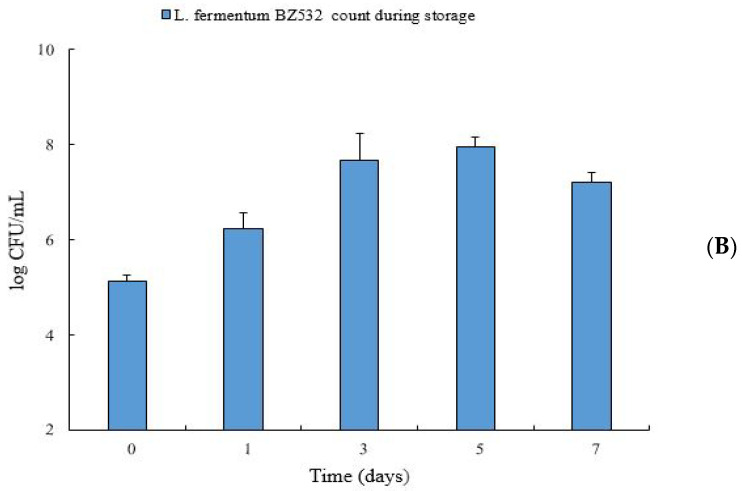
Changes in (**A**) TVC of bozai treatments and (**B**) LAB enumeration during storage of 7 days at 4 °C. Data with different letters represent significant differences (*p* < 0.05).

TVC is extensively used as a key parameter to evaluate the general quality status of the fermented product of any origin. Changes in TVC of bozai are presented in Figure 2a. TVCs of two bozai treatments (BZ-*C* and BZ-*Lf*) were 4.8 and 4.43 log CFU/mL, respectively, at day 0. All bozai samples were evaluated throughout 7 days of storage at 4 °C and the control group of bozai (BZ-*C*) treatment showed a steady increase, BZ-*C* expressed the highest value, and BZ-*Lf* showed the minimum as compared with the BZ-*C* group. The TVCs of BZ-*C* reached 7.22 log CFU/mL on day 7; instead, BZ-*Lf* showed 5.03 log CFU/mL, relatively lower than the control group of bozai, indicating that the bacteriocinogenic strain with the antimicrobial product had a substantial inhibition effect on TVC of bozai, which followed the findings of previously studies [35,36]. The microbiological standard of fermented food products was standardized at 6 log CFU/mL [37]; according to standardized values, the results of BZ-*C* bozai exceed that limit, but accordingly, BZ-*Lf* is still edible. These results exhibited that the antimicrobial product could prolong the shelf-life of bozai by inhibiting the viable count of bacteria.

*L. fermentum* BZ532 counts in bozai beverage during 7 days of storage at 4 °C are shown in Figure 2b. The count level increased from 5.11 to 7.95 log CFU/mL in the first five days of storage at 4 °C, then gradually decreased to 7.19 log CFU/mL) after 7 days. The evolution of the lactic acid strain level in bozai is dependent on storage time and temperature, as previously observed by other authors [38]. These microorganisms have played a substantial role in food fermentations, leading to texture and flavor change together with a preservative effect, increasing the shelf-life of the altered product [17].

### 3.2. Co-Culturing of L. fermentum BZ532 with Pathogenic Bacteria during Bozai Production

**Figure 3:**
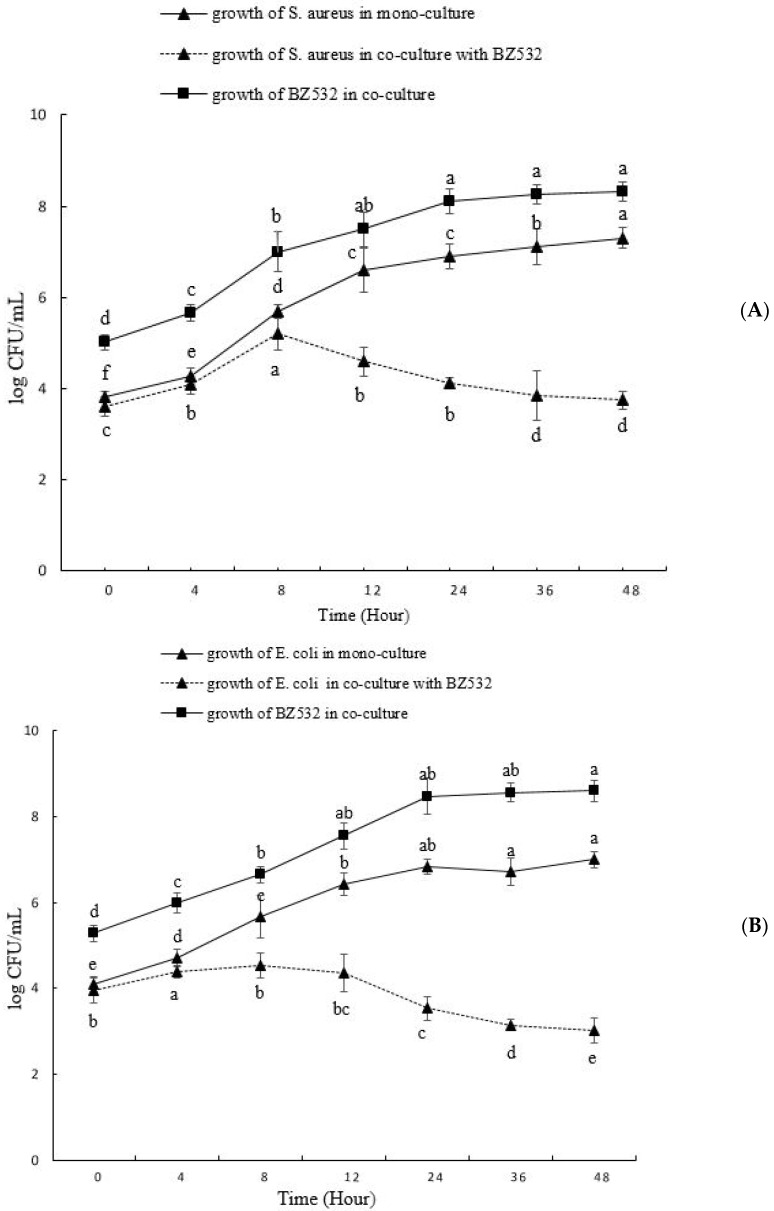
Co-culturing of *L. fermentum* BZ532 strain against pathogenic strains (**A**) *S. aureus* and (**B**) *E. coli* in bozai treatments during 48 h fermentation. Data with different letters represent significant differences (*p* < 0.05).

The bacteriocin LF-BZ532 produced by the strain *L. fermentum* BZ532 has been preliminarily characterized in a previous study [27]. The inhibition spectrum of BZ532 was assessed by co-culturing the BZ532 strain against pathogenic strains such as *S. aureus* ATCC6538 and *E. coli* k-12 separately. To test the ability of *L. fermentum* BZ532 as a protective culture, fresh bozai BZ-*LF* was inoculated simultaneously with indicator pathogen strains cited above. For control purposes, mono-cultures of bozai BZ-*C* with *S. aureus* ATCC6538 and *E. coli* k-12 were also performed separately. *S. aureus* ATCC6538 presented a prompt growth rate from 3.82 to 7.11 log CFU/mL during a mono-culture of 48 h. Meanwhile, the growth rate of *S. aureus* ATCC6538 during co-culturing with *L. fermentum* BZ532 showed a slight rise, then significantly (*p* < 0.05) reduced to a level of 3.85 log CFU/mL within 24 h of fermentation, as stated in Figure 3a. Microbial growth of *E. coli* was increased 2–3 log CFU/mL during monoculture in bozai; however, a limited increase was observed during co-culturing from 3.95 to 4.53 log with bacteriocinogenic *L. fermentum* BZ532 during the initial 8 h (Figure 3b), then declining gradually afterward. However, no effect on *L. fermentum* BZ532 growth was observed during co-culturing. Similar results were previously described [35,39].

A significant decrease in the growth of two indicator strains was detected in co-cultures with *L. fermentum* BZ532, and food spoilage strains were inhibited during bozai fermentation. The antimicrobial peptides produced during fermentation accumulated continuously, leading to increased antimicrobial potential. A comprehensive mechanism is needed to validate this in further studies. These results recognized that *L. fermentum* BZ532 can used as a protective culture in bozai to limit the growth of pathogenic food spoilage microbes.

### 3.3. In Situ Bacteriocin Activities in Bozai during Storage

**Figure 4:**
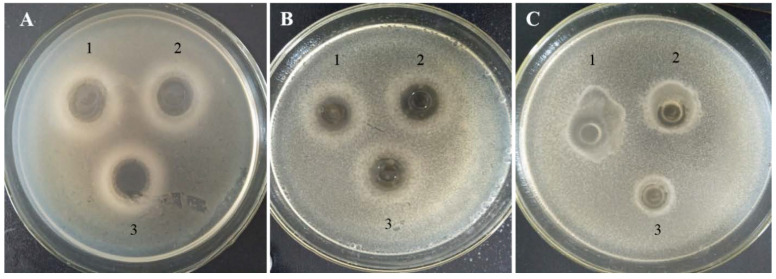
Antimicrobial action of in situ production of bacteriocin in bozai BZ-*LF* against (**A**) *S. aureus*, (**B**) *E. coli* k-12, and (**C**) *Salmonella* sp. D104. 1, 2, 3 represents as triplicate of inhibition zone (*n* = 3).

The application of bacteriocinogenic strains as a protective culture is based on in situ production of bacteriocins in several reported studies [30,40]. CFS extracted from bozai treatments showed antimicrobial activities, the neutralized CFS of bozai BZ-*LF* treatment showed a wide range inhibitory spectrum against *S. aureus* ATCC6538 and *E. coli*, but not against *Salmonella* sp. D104, as presented in Table 1. In Figure 4, this was varied from LF-BZ532 produced by *L. fermentum* BZ532 in MRS (broth) medium, which expressed strong inhibition against *Salmonella* sp. D104 [27]. The production of bacteriocins might be intensively affected by the composition of the growth medium [16]. Bozai treatment with bacteriocinogenic strain inoculation presented in situ bacteriocin production against two pathogens, as stated previously. However, generally, LAB can produce different organic products such as organic acids (lactic acid, propionic acid, acetic acid, and so on), acetaldehyde, diacetyl, and bacteriocins with antimicrobial characteristics [41]. Bacteriocins are ribosomally synthesized antimicrobial peptides/proteins, showing a diverse activity spectrum. Several bacteriocins produced by LAB strains have been documented and proved to be effective against *S. aureus*, *L. monocytogenes*, *Bacillus* sp., *Clostridium* sp., and other spoilage microbes [42]. Numerous LAB strains from cereal-based products have been recognized as a good producer of antimicrobial compounds against different foodborne pathogens; i.e., *E. coli*, *L. monocytogenes*, and *S. aureus*, among others [43].

Based on this study, *L. fermentum* BZ532 can inhibit the growth of *E. coli* and *S. aureus* in the fresh bozai production system thanks to its bacteriocinogenic ability during storage; however, it was not able to counter the activity of *Salmonella* sp. in fresh bozai. Further studies must be directed to evaluate the inhibitory spectrum of *L. fermentum* BZ532 bacteriocin at various concentrations with advanced methodology.

### 3.4. Changes in Physicochemical Parameters of Bozai during Storage

The effect of bacteriocinogenic activity and storage on pH and acidity is presented in Table 2. pH and titratable acidity are inversely correlated parameters. Moreover, the pH of bozai samples declined, while the total acidity percentage indicated a significant (*p* < 0.05) increase during fermentation and storage, as presented in Table 2, with initial pH and acidity between 5.75 and 4.51 and 0.89% and 2.37%, respectively, in the control BZ-*C* and BZ-*Lf* group of bozai. The pH decreased slightly from 5.75 to 5.11 in the control group of bozai (BZ-*C*), but a significant decline from 4.51 to 3.11 in the BZ-*LF* group was observed during 7 days of storage at 4 °C. As pH and titratable acidity are inversely correlated, the initial acidity of BZ-*LF* was 2.37%; thereafter, it increased to 3.88%. BZ-*Lf* treatments were also observed in terms of their acidity percentage changes during storage and showed a significant increase in acidity level during 7 days of storage. A significant (*p* < 0.05) decrease in pH of bozai samples was due to the specific microbiological activity of *L. fermentum* BZ532 in terms of their lactic acid production by glycolysis of various carbohydrates during fermentation and storage. The findings are compatible with previously reported studies that were targeted to optimize different cereal-based probiotic food products [44,45,46,47]. Food formulations with pH levels (3.5–4.5) are desired because they boost the stability and benefits of probiotic microbes [48] as well as inhibit the growth of coliforms [49].

The changes in protein content of bozai with the effect of bacteriocinogenic strain are presented in Table 2. The maximum protein content of the control bozai BZ-*C* group was 3.26%; however, BZ-*Lf* had 3.18%. It is indicated that no significant (*p* < 0.05) effect of the bacteriocinogenic strain was explored on the protein content, as no consistent changes in total protein content were observed during fermentation and storage.

### 3.5. Changes in Organoleptic Properties of Bozai during Storage

The effect of bacteriocinogenic strain and storage days on sensory attributes of bozai is presented in Table 3. It was determined that storage time and antimicrobial compounds had a significant (*p* < 0.05) effect on the organoleptic characteristics (color, odor, mouthfeel, and overall acceptability) of bozai. As likely, a decline in sensory scores of bozai treatments during storage was exhibited, demonstrating a slight decrease in overall acceptability. All of the bozai treatments exhibited maximum sensory scores at storage day 1, with a decrease during storage of 7 days, and indicated that antimicrobial products did not have any adverse effect on bozai quality at day 1. The highest sensory score of the BZ-*Lf* bozai group at day 1 in terms of color was 6.55, while the odor and mouthfeel score was 6.33 and 6.63, respectively, as presented in Table 3. The highest overall acceptability score of bozai BZ-*Lf* was 6.66; however, the control group of bozai BZ-*C* showed the lowest overall acceptability of 5.60, a clear indication of the significant (*p* < 0.05) effect of bacteriocinogenic strain with antimicrobial compounds on bozai treatments of BZ-*Lf*. The results of various sensory parameters revealed that the bacteriocinogenic strain with bacteriocin could positively affect the overall quality of bozai during storage of 7 days. In conclusion, these findings presented that consumers may accept LAB bozai as a natural functional cereal beverage.

## 4. Conclusions

A bozai drink was developed by inoculation of *L. fermentum* BZ532 and compared with a control group of bozai with natural starter (Sourdough) inoculation. *L. fermentum* BZ532 showed significant antimicrobial activity against foodborne pathogens such as *S. aureus* and *E. coli* during the co-culturing study, and in situ production of antimicrobial compounds also had an antimicrobial effect on two food spoilage microbes, i.e., *S. aureus* and *E. coli*. The microbiological status of bozai treatments concerning TVC and BZ532 count was also determined during storage. Bozai treatments (BZ-*C* and BZ-*LF*) were also subjected to physicochemical characteristics in terms of pH, titratable acidity, and protein content. Overall acceptability of bozai treatments was also determined by evaluating several organoleptic parameters, and it was found that the BZ-*LF* bozai had significantly high acceptability by the expert panelists.

## Figures and Tables

**Table 1:** In situ antimicrobial activities of prepared bozai treatments.

Indicator Strains	Antimicrobial Activities ^1^					
	24 h		36 h		48 h	
	BZ-*C*	BZ-*LF*	BZ-*C*	BZ-*LF*	BZ-*C*	BZ-*LF*
*Staphylococcus aureus*	1.66 ± 0.21 ^b^	3.32 ± 0.08 ^a^	1.81 ± 0.17 ^b^	4.85 ± 0.10 ^a^	1.93 ± 0.15 ^b^	5.54 ± 0.13 ^a^
*Escherichia coli* k-12	1.84 ± 0.15 ^b^	3.64 ± 0.06 ^a^	1.98 ± 0.17 ^b^	5.36 ± 0.13 ^a^	2.09 ± 0.22 ^b^	5.64 ± 0.09 ^a^
*Salmonella* sp. D104	1.02 ± 0.16 ^a^	0 ± 0 ^b^	1.11 ± 0.13 ^a^	0 ± 0 ^b^	1.26 ± 0.21 ^a^	0 ± 0 ^b^

Data with different letters represent significant differences (*p* < 0.05). ^1^ Antimicrobial activities: diameter of inhibition zones (mm); values expressed as means ± SD (*n* = 3).

**Table 2:** Changes in physicochemical parameters of Bozai samples during storage of 7 days at 4 °C.

Parameters	Treatments	Storage Time (Days)			
		1	3	5	7
pH	BZ-*C*	5.75 ± 0.13 ^a^	5.47 ± 0.08 ^a^	5.22 ± 0.09 ^a^	5.11 ± 0.11 ^a^
	BZ-*Lf*	4.51 ± 0.11 ^b^	3.85 ± 0.10 ^b^	3.25 ± 0.06 ^b^	3.11 ± 0.06 ^b^
Total acidity (%)	BZ-*C*	0.89 ± 0.08 ^b^	1.36 ± 0.04 ^b^	1.70 ± 0.08 ^b^	1.89 ± 0.07 ^b^
	BZ-*Lf*	2.37 ± 0.13 ^a^	3.14 ± 0.04 ^a^	3.69 ± 0.14 ^a^	3.88 ± 0.07 ^a^
Protein (%)	BZ-*C*	3.26 ± 0.41 ^b^	2.82 ± 0.38 ^b^	3.15 ± 0.31 ^b^	2.69 ± 0.21 ^b^
	BZ-*Lf*	3.07 ± 0.51 ^a^	2.99 ± 0.23 ^a^	3.18 ± 0.33 ^a^	2.87 ± 0.26 ^a^

Results expressed as means ± SD (*n* = 3). Data with different letters represent significant differences (*p* < 0.05).

**Table 3:** Changes in organoleptic properties of Bozai samples during storage of 7 days at 4 °C.

Parameters	Treatments	Storage Time (Days)			
		1	3	5	7
Color	BZ-*C*	5.76 ± 0.40 ^b^	4.88 ± 0.32 ^b^	5.1 ± 0.17 ^b^	4.66 ± 0.23 ^b^
	BZ-*Lf*	6.55 ± 0.23 ^a^	5.70 ± 0.20 ^a^	5.93 ± 0.40 ^a^	5.53 ± 0.50 ^a^
Odour	BZ-*C*	4.26 ± 0.46 ^b^	4.56 ± 0.40 ^b^	4.66 ± 0.57 ^b^	5.66 ± 0.57 ^b^
	BZ-*Lf*	6.33 ± 0.57 ^a^	5.56 ± 0.51 ^a^	6.50 ± 0.5 ^a^	6.83 ± 0.76 ^a^
Mouthfeel	BZ-*C*	5.26 ± 0.64 ^b^	5.13 ± 0.80 ^b^	4.86 ± 0.80 ^b^	4.56 ± 0.51 ^b^
	BZ-*Lf*	6.63 ± 0.47 ^a^	5.86 ± 0.51 ^a^	6.46 ± 0.45 ^a^	5.66 ± 0.57 ^a^
Overall acceptability	BZ-*C*	5.60 ± 0.36 ^b^	5.5 ± 0.86 ^b^	5.5 ± 0.5 ^b^	5.20 ± 0.34 ^b^
	BZ-*Lf*	6.66 ± 0.76 ^a^	6.06 ± 0.4 ^a^	6.0 ± 0.30 ^a^	5.93 ± 0.40 ^a^

Results are expressed as means ± SD (*n* = 3). Data with different letters represent significant differences (*p* < 0.05).

## Data Availability

Data is contained within the article.
